# A Review of the Forward Problem in Electrocardiographic Imaging

**DOI:** 10.3390/jimaging12060224

**Published:** 2026-05-25

**Authors:** Xiafeng Zhang, Xuanhe Han, Kaiyu Chen, Yucheng Wang, Wei Li, Shaoxi Wang

**Affiliations:** School of Microelectronics, Northwestern Polytechnical University, Xi’an 710072, China

**Keywords:** electrocardiographic imaging, forward problem, cardiac surface potential model, equivalent double-layer model, transmembrane voltage model

## Abstract

Electrocardiographic imaging (ECGI) is a noninvasive technique for reconstructing cardiac electrical activity by recording body-surface potentials and geometric information of the heart and torso. The ECGI forward problem is the cornerstone of ECGI. Depending on the forward model, ECGI can reconstruct epicardial/endocardial surface potentials, activation/recovery sequences, transmembrane voltages, and other electrophysiological quantities of interest. This article reviews the modeling process and research progress of forward modeling. This review systematically summarizes the mathematical methods used in the ECGI forward problem by classifying them into three representative models: the cardiac surface potential (CSP), equivalent double-layer (EDL), and transmembrane voltage (TMV) models, with detailed derivations and guidance on their practical applications.

## 1. Introduction

**Figure 1:**
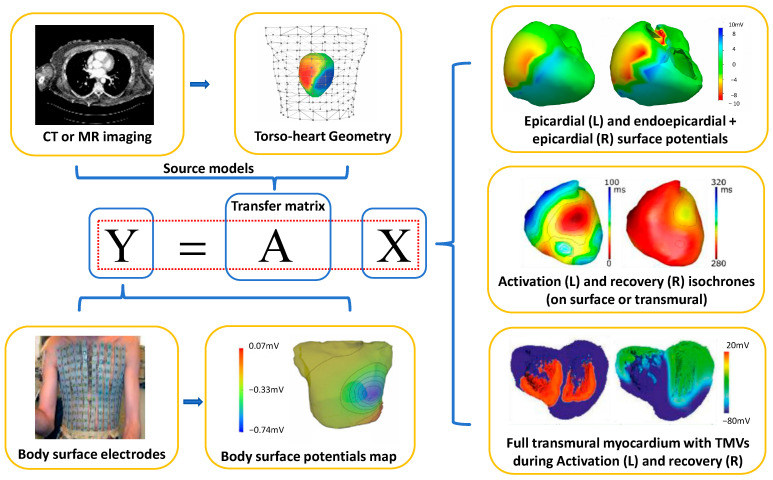
ECGI problem. ECGI reconstructs the electrical properties of the heart by recording the body-surface potential and the torso–heart geometry obtained from computer tomography (CT) or magnetic resonance (MR) imaging. The electrical properties of the reconstructed heart depend on the source model of the heart, which can be cardiac surface potential, activation/recovery time, or transmembrane voltage [26,29,30].

Electrocardiographic imaging (ECGI) is a noninvasive approach for reconstructing cardiac electrical activity. It achieves goals by using body-surface potential recordings combined with patient-specific heart–torso geometries. ECGI is notable for its noninvasive nature, high resolution, and capability of continuous mapping [1]. Through this technique, details such as extracellular electrical potentials on both endocardial and epicardial membranes [2,3], the activation time sequence of the cardiac surface [4,5], and cardiac transmembrane voltage [6,7,8] can be discerned, as shown in Figure 1.

To improve the transparency and reproducibility of this review, a structured literature search was conducted to identify studies related to the ECGI forward problem. Publications from the early development of cardiac source modeling in the 1970s to 2026 were searched in PubMed, Web of Science, IEEE Xplore, and Scopus. Search terms included combinations of “ECGI forward problem”, “electrocardiographic imaging forward model”, “cardiac surface potential”, “equivalent double layer”, “transmembrane voltage”, “cardiac electrical source model”, “body surface potential mapping”, and “thoracic volume conductor”. Additional relevant studies were identified from the reference lists of highly cited and methodologically important articles.

Studies were included if they directly addressed ECGI forward modeling and provided mathematical formulations, computational implementations, experimental validation, or clinically relevant applications of cardiac-to-torso electrical mapping. Studies focusing only on inverse reconstruction, abstracts or non-peer-reviewed materials, and papers lacking sufficient methodological detail were excluded. After screening titles, abstracts, and full texts, 573 publications met the eligibility criteria. After further synthesis and categorization, 171 representative publications were selected and retained in the review. Since as early as the 1960s, numerical methods have been used to solve the electrocardiography forward problem of arbitrary shape volume conductors [9,10,11]. In 1978, Miller and Geselowitz employed a multi-dipole simulated electrocardiogram to detail the activation sequence and cardiac action potential of a healthy heart, in the context of infarction and ischemia [12,13]. Fast forwarding to 1998, Hren et al. demonstrated the role of fiber orientation in a three-dimensional propagation model of real human ventricular myocardium [14]. Over the past half century, the evolution of ECGI technology has been remarkable. It progressed from initial analytical studies [15,16] to torso volume conductors [17,18,19,20], extensive large animal model testing [21,22], and ultimately human application [23,24,25]. Presently, ECGI stands as an indispensable tool, demonstrating its significance in both scholarly research and clinical practice [26,27,28]. Together, these advances established the forward operator that mapped cardiac sources to body-surface potentials and, in turn, laid the foundation for ECGI; ECGI inverts this operator to estimate cardiac activity from body-surface potentials. Building on this forward operator, ECGI addresses the inverse problem: given measured body-surface potentials Y, estimate cardiac sources X through the linear model:

(1) Y=AX+ε

where A denotes the forward operator, or transfer matrix, that maps cardiac sources to body-surface potentials, and ε denotes the residual vector of the model.

Usually, the inverse problem of ECGI does not have a unique solution and is ill-conditioned [31], mainly because multiple cardiac electrical activity patterns can lead to similar surface potentials. Consequently, small perturbations in the data may yield large reconstruction errors and impractical solutions [32,33]. To obtain stable and clinically meaningful estimates, additional regularization constraints are required. Over recent decades, a wide range of regularization strategies and optimization methods have been developed and rigorously evaluated [34,35], with approaches informed by physical and physiological priors providing notable gains in stability and robustness [36]. Zhang et al. reported that, under specific torso-tank experimental conditions, ECGI reconstruction was relatively robust to moderate rigid cardiac motion, with limited changes in correlation coefficient for 10 mm translations and 10° rotations [37].

The transfer matrix A is derived from the ECGI forward problem, which computes body-surface potentials for a specified source representation (e.g., epicardial/endocardial potentials or transmembrane voltages) within a patient-specific heart–torso geometry and conductivity model. In compact form, the forward problem defines the mapping from cardiac sources X to measured potentials Y. This forward operator is foundational: modeling choices in the forward problem—source representation, geometry/segmentation, tissue anisotropy, and boundary conditions—determine A and thereby the identifiability, conditioning, and ultimate performance of the inverse reconstruction.

Accordingly, this review aims to:

1. Summarize recent advances in ECGI forward modeling.
2. Detail three canonical forward models—the cardiac surface potential (CSP), equivalent double-layer (EDL) and transmembrane voltage (TMV) frameworks—derive the corresponding transfer matrices, and discuss their advantages, limitations, and open challenges.
3. Outline future directions in forward modeling to further advance ECGI.

## 2. Modeling of the ECGI Forward Problem

**Figure 2:**
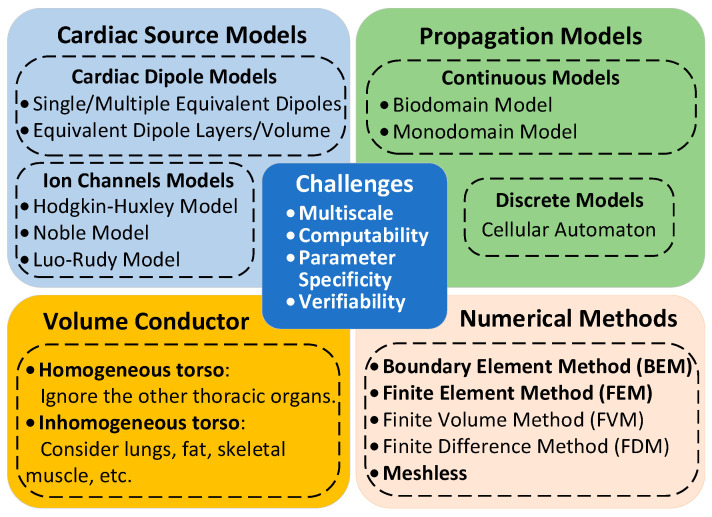
Modeling methods of the ECGI forward problem.

The ideal forward solution, which can provide an accurate correspondence between cardiac sources and body-surface potentials, encompasses data ranging from the intricacies of ion-channel proteins to the expansive human thoracic cavity, spanning timescales from nanoseconds to minutes or even hours. However, achieving this in a clinical setting seems daunting. To get workable solutions, researchers simplify and approximate, sacrificing some detail and precision. This section thus offers a brief overview of the forward-problem modeling from various angles, helping understand how to balance complexity and practicality, as shown in Figure 2.

### 2.1. Cardiac Bioelectricity: From Micro to Macro Perspectives

The forward solution describes an outward mapping from prescribed cardiac electrical sources to the resulting potentials on the myocardium and the torso (i.e., body-surface potentials) [38]. To effectively address this challenge, researchers must first understand the origins of cardiac bioelectricity and their connections to remote manifestations of bioelectricity. This complex scenario represents a multiscale electrophysiological modeling challenge that can be approached from three distinct perspectives: micro, transition and macro [33].

From the cellular perspective, the modeling methods prioritize the cellular level. It captures the dynamic shifts of ion channels on cell membranes, the equilibrium of ions within and outside cells and the action potential of cardiomyocytes. Pioneering works in this field include Hodgkin and Huxley’s creation of the world’s first computational cellular model, which laid the foundation for understanding the electrical activity of cells at the molecular level [39]; Denis Noble’s first computational cardiomyocyte model, which further advanced the study of cardiac cell electrophysiology [40]; and Beeler and Reuter’s action potential simulation for ventricular myocytes, which provided insights into the electrical behavior of ventricular cells [41]. These foundational models paved the way for further research, leading to the emergence of advanced models such as the Luo–Rudy model [42,43] for studying the action potential of ventricular cardiomyocytes and the Nygren model for atrial myocytes [44].

The transition layer serves as an intermediary, linking microscopic and macroscopic models. It encapsulates the heart’s fibers and tissues. At its core, the myocardial fiber model shows that numerous myocardial cells adhere to cardiac muscle fibers, allowing the electrical excitation produced by cells to conduct along the direction of the fibers. The tissue model, which resembles a grid structure, is a combination of multiple fiber-level models. This configuration effectively simulates electrical excitation propagation from a localized to a more global scope. Essentially, the transition layer offers a quantitative analysis of the nexus between ion-channel alterations at the microscopic layer and the propagation of electrical excitation at the macroscopic heart level. Techniques like Huygens’ principle [45] or the model based on the reaction–diffusion equation [46] facilitate the creation of two- or even three-dimensional heart potential propagation models.

Macroscopic electrophysiological models encompass 3D representations at either the cardiac organ or torso level. Based on comprehensive anatomical data of the heart and torso, researchers can compute body-surface potentials spatially based on the source, propagation, and distribution of cardiac electrical excitations [47]. A salient advantage of this macroscopic model is its ability to provide an intuitive representation of macroscopic functional transformations due to physical modifications in the microlayer, thus probing the link between arrhythmia manifestations and microscopic electrophysiology.

For practical forward solution, it is imperative to elucidate the source and its propagation. Additionally, the “volume conductor”, or the space housing the source, demands detailed understanding. Given the model’s intricacy, its computational feasibility becomes paramount. This section succinctly explores the forward solution from the perspectives of the cardiac source model, the propagation mechanism, the volume conductor, and the numerical method.

### 2.2. Cardiac Source Models

The ECG is a result of the action potential generated by the rhythmic excitation and contraction of myocardial cells. At its core, the ECG represents the periodic flux of ions (e.g., K+, Na+, etc.) across the myocardial cell membrane. Central to our understanding of this process is the Hodgkin–Huxley model [39]. This foundational model, which was among the first to quantify the properties of cell transmembrane potentials and ion channels, remains pivotal to contemporary cell and membrane modeling. Its significance is well-documented in numerous review articles and textbooks [48,49]. Based on the Hodgkin–Huxley model, the action potential of an individual cardiomyocyte can be articulated through the following general equation:

(2) $$C_{M}\frac{\partial V}{\partial t}=-(I_{ion}+I_{stim})$$

where $C_{M}$ is the membrane capacitance, V is the membrane potential, $I_{ion}$ is the sum of the active ionic currents, and $I_{stim}$ is the externally applied stimulus current.

Since the introduction of the first cardiac membrane model by Noble et al. in 1962 [50], there have been numerous adaptations that are tailored to various ion channels and different target animal species [48,51]. Among these, the model presented by Luo Rudy [43] stands out due to its widespread use. Additionally, Schroder et al. reviewed the effects of the cardiomyocyte circadian clock in ion-channel regulation and cardiac electrophysiology [52]. Xinwei et al. provide a comprehensive overview of the membrane simulation models, encapsulating both electrical and mechanical perspectives [53].

Addressing the forward problem of ECGI often entails breaking down the heart into hundreds of thousands, or even millions, of minuscule elements. Each of these elements has its own distinct membrane model. Given the computational demands inherent to this approach, the selection of a membrane simulation model is critical for computational tractability, since detailed ionic models introduce numerous stiff state variables, which necessitate extremely small timesteps and require large memory for meshes with millions of elements. Thus, identifying a streamlined and efficient ion-channel model is paramount. Current ECGI solutions commonly adopt simplified cardiac source models, such as the dipole model.

One of the most straightforward and renowned methods to characterize the heart’s electrical activity is through the cardiac dipole. In many foundational ECGI models, the heart’s source is approximated either as a single or multiple equivalent dipoles [54] or as equivalent dipole layers [55,56,57]. While the single dipole is intuitively simple and computationally efficient, it provides only a rudimentary representation of the heart’s electrical activity. The dipole is an inadequate model, so it is rarely considered in contemporary research, but it still has the largest impact on clinical education and interpretation of the ECGI [49,58]. The dipole source model is discrete, starting with the expression of the potential of a single dipole in infinite space:

(3) $$\phi =\frac{1}{4\pi \sigma }\frac{\vec{r}}{r^{3}}\cdot \vec{D}$$

where $\vec{r}$ is the vector from the dipole position to the spatial position where ϕ is calculated, and $\vec{D}$ is the dipole moment that specifies the direction and magnitude of the current dipole source.

In an unbounded homogeneous isotropic bulk conduction medium with an electrical conductivity σ, the potential ϕ at the observation point can be equivalently expressed as a superposition of dipole fields. A volume distribution of dipole sources can be described by the density function $\vec{M_{V}}$, which is similar to the volume charge density, where $\vec{M_{V}}$ is the dipole moment per unit volume and has dimension Am^−1^. The total potential field is obtained by integrating the biological source in domain V:

(4) $$\phi =\frac{1}{4\pi \sigma }\int_{V}\vec{M_{V}}\cdot \frac{\vec{r}}{r^{3}}dV$$

Through the combination of dipoles, many other forms of discrete sources can be formed, some of which prove useful in very special cases [59]. For more details on discrete models, see Gulrajani’s review [49,60].

### 2.3. Propagation Models

To simulate a complete heartbeat, dynamic bioelectric sources that evolve over time are essential. The most common method to produce such a time-varying source involves simulating the propagation of excitation within tissue. The simulation consists of two primary phases, with various approaches [49]. The first phase is to predict the sequence of depolarization across different tissue regions. This results in determining activation and recovery timelines, signifying the actual diffusion of excitation. The second phase is to calculate potentials either within the tissues or predominantly on the epicardium and endocardium using the timelines.

A frequently adopted approach to calculate potential involves synchronizing different heart regions by portraying them as dipole sources. This representation uses an activation wave front to define their orientation and respective activation/repolarization times. Alternatively, these sources can be depicted as moving double layers, where their trajectory and timing are dictated by propagation parameters. Instead of directly estimating the excitation diffusion, another approach is to leverage pre-established knowledge of this sequence, which may be derived experimentally, and then extrapolate extracardiac potential based on this data.

In discrete dipole models, numerous foundational studies have employed cellular automata to address propagation challenges [61,62]. The cellular automaton technique segments the electrical wave propagation problem into two components: a regular grid and an automaton. While the grid specifies the size and topology of the target domain, the automaton, which is driven entirely by a set of internal rules, embodies the behavior of a single cell. The main merit of cellular automata is computational efficiency, and the entire heart simulation is possible without resorting to costly super computing resources. Nevertheless, this approach has its limitations. In most implementations, the grid’s units surpass the dimensions of a real cardiac cell, causing the resulting wave front’s shape to be influenced by grid topology. Moreover, the cellular automata approach is not ideal for simulations focusing on dynamic cell activities, like ischemia occurrences, since it excludes genuine cellular electrophysiology and, hence, cannot adapt to most external stimuli [49,63].

In continuous models, the bidomain model is one of the most effective methods for describing the propagation of myocardial electrical activity [64,65]. The bidomain model represents heart tissue as a syncytium, consisting of both intracellular and extracellular domains. The intracellular domain accounts for all intracellular space, while the extracellular domain represents all extracellular space; both share the same physical space within the myocardium. A cell membrane, with no volume in the traditional bidomain model, separates these two overlapping domains, yet it is uniformly distributed throughout the tissue. The membrane is embedded with ion channels that regulate charge distribution and current flow, which in turn produce action potentials within the cells.

The monodomain model is a simplification of the bidomain model, making the numerical solution more tractable and highlighting its utility [66]. Studies [67] indicate that the trajectories of the spiral wave tip computed under both the monodomain and bidomain models are nearly identical. Remarkably, there is no discernible difference between the monodomain model and the bidomain model in terms of single-cell action potential propagation without an applied current, even when considering the anisotropy of non-uniform conduction in the extracellular region [68]. However, in certain pathological conditions, such as defibrillation, the significance of non-uniform anisotropy in both intracellular and extracellular regions cannot be overlooked.

FitzHugh and Nagumo were the pioneers in developing a monodomain model derived from a two-variable cellular model, which is also known as the FitzHugh–Nagumo model [69,70]. The mathematical formulation of this model is given by

(5) $$\begin{array}{l} \frac{du}{dt}=\nabla (D\nabla u)+f_{1}(u,v),\\ \frac{dv}{dt}=f_{2}(u,v) \end{array}$$

where u is the dimensionless excitation variable, which can be identified with transmembrane potential, v is the dimensionless recovery variable, and D is the diffusion tensor. In this reaction–diffusion system, the diffusion tensors and parameters remain constant over time, but they may vary across space. The passive diffusion of current is controlled by the diffusion term ∇(D∇u) in the first equation, whereas the ionic currents are represented by the two subsequent terms $f_{1}(u,v)$ and $f_{2}(u,v)$. Variations of $f_{1}(u,v)$ and $f_{2}(u,v)$ produce different action potential shapes.

The FitzHugh–Nagumo model characterizes not only the excitation of cardiac sources but also their propagation. The FitzHugh–Nagumo method has gained popularity primarily because of its exceptional computational efficiency in simulating activation and recovery. Numerous studies have demonstrated its efficacy, particularly in simulating reentrant waves, which represent arrhythmias [6,7,71,72].

### 2.4. Volume Conductor and Tissue Conductivity

Electrical signals generated by the heart are transmitted to the body’s surface via torso conduction. This transmission raises questions regarding the impact of torso inhomogeneity on ECG signals. Bear et al. assessed the precision of various forward models [18], particularly focusing on the influence of the torso’s inhomogeneous volume conductor [73]. During sinus rhythm in anesthetized pigs with closed chests, body-surface and epicardial potentials, along with epicardial and endocardial ventricular pacing, were recorded simultaneously. Two torso model types were evaluated: a homogeneous, isotropic model and an inhomogeneous model that explicitly represents the lungs, subcutaneous fat, and anisotropic skeletal muscle. In their research, reference values include approximately 0.07 S/m for lung, 0.67 S/m for blood, 0.4 S/m for myocardium, 0.44 S/m for skeletal muscle, and lower values for fat and bone, although these values vary across studies. These conductivities enter the passive torso volume-conductor equation as scalar conductivity distributions or, for anisotropic tissues such as skeletal muscle, as conductivity tensors. Consequently, they directly affect the forward operator that maps cardiac sources to body-surface potentials. Bear et al. compared homogeneous and inhomogeneous torso models using simultaneous epicardial and body-surface recordings in closed-chest pigs and showed that homogeneous models introduced substantial spatial inaccuracies, including errors in potential extrema and attenuation patterns. Incorporating lungs, anisotropic skeletal muscle, and subcutaneous fat reduced, but did not fully remove, these discrepancies. Therefore, tissue-conductivity assignment should be considered an important factor in ECGI forward modeling rather than a purely technical parameter.

Their predictions were compared with the measured body-surface potential. Discrepancies between the simulated and measured potentials were attributable primarily to the voltage referencing and to peak-amplitude scaling, not to inaccuracies in the torso–heart geometric model. While the utilization of inhomogeneous models can minimize the disparities between measured and predicted potential distributions, it does not eradicate them entirely. This observation aligns with a prior study conducted on dogs by Ramsey et al. [21].

In a clinical setting, certain conductivities, such as high lung conductivity in pulmonary edema and low conductivity in conditions like cystic fibrosis, cannot be noninvasively determined for patients [17]. Undoubtedly, considering torso volume conductors as electrically homogeneous streamlines the clinical application of ECGI. Compared with a homogeneous torso model of identical geometry, inhomogeneity influences the body-surface potential’s value, albeit exerting a minimal effect on the body-surface potential pattern. Such a characteristic holds significance in clinical applications, as the majority of ECG diagnostic standards primarily hinge on electrocardiogram morphology, rather than absolute voltage magnitudes. Ramanathan et al. suggested that, in their ECGI inverse-reconstruction study using a dog heart suspended in a human-shaped torso-tank model, homogeneous torso models produced only slightly less accurate reconstructions than inhomogeneous models for epicardial potentials, electrograms, isochrones, and pacing-site localization. Therefore, under these specific experimental conditions and target endpoints, torso inhomogeneities may have a limited influence on noninvasive epicardial reconstructions for clinical ECGI applications [20]. However, the generalizability of this conclusion to broader ECGI applications remains to be further validated.

### 2.5. Comparative Overview of Numerical Methods for Solving the Forward Problem

The appropriate numerical method often depends on the specific forward model in use, and there is no universally optimal method that suits all applications [31]. The choice among different numerical methods can be influenced by factors such as the type of source model, mode of propagation, assumptions inherent to the volume conductor, and the expected simulation results. The torso–heart geometry used in the forward problem is usually processed from CT/MRI images [74]. While these classical numerical techniques are prevalent across various scientific and engineering fields, they find particular applicability in addressing diverse forward problems. Here, we offer a concise overview of these prevalent numerical methods for forward problems.

**Boundary Element Method (BEM):** BEM segments only the domain boundaries, so geometry edits and parameter sweep require remeshing and reassembly only on surfaces rather than the full volume. In practice, this means faster updates for (i) moving or adding electrodes, (ii) local refinement near sharp anatomical features, (iii) patient-specific torso/heart boundary changes, and (iv) modeling unbounded exterior domains—capabilities that are more cumbersome with volume-meshed finite element/volume methods [75]. Given its focus solely on surface boundary conditions, the matrix associated with BEM in forward problems tends to be significantly more compact than those in other methods. Historically, the BEM boasts the richest lineage in cardiac bioelectricity applications. It stands as a predominant method in the ECGI forward problem, having been a staple in both early simulation studies and numerous contemporary studies [76,77]. The majority of forward and inverse problems in the ECGI, especially those centered on epicardial potential, have employed BEM [78,79]. The widespread adoption owes much to pioneering studies by Barr et al. [80]. In the ECGI forward problem, BEM is particularly apt when the torso consists of multiple uniform isotropic regions, each with a distinct conductivity value. However, the BEM is less ideal for anisotropic regions [31].

**Finite Element Method (FEM):** The FEM operates by decomposing the entire domain into a grid, morphing the torso into an assembly of continuous volume elements characterized by basic geometries, such as tetrahedrons or hexahedrons. Each of these elements represents a fundamental component of the overarching problem. When juxtaposed with BEM, the FEM’s mesh showcases a heightened intricacy and exhibits increased sensitivity to mesh configurations. Any alterations in the heart or torso’s geometry demand meticulous construction of the mesh, encompassing nodes and polygons. The strength of FEM lies in its versatility: it can adeptly handle anisotropic tissue zones and accommodate various conductivity profiles within the torso. Colli-Franzone et al. are likely the pioneering contributors to the FEM literature [81], and subsequent research has extensively employed FEM to address an array of ECG challenges [82,83,84]. Similar approaches include the Finite Volume Method (FVM) [84,85,86,87] and the Finite Difference Method (FDM) [31,88], which have also garnered favor in addressing the cardiac bioelectrical quandaries.

**Meshless:** The meshless method is characterized by its sole need for boundary descriptions and node distribution, eliminating the requirement for model element connections and grid subdivisions [89,90]. This leads to a convenient node configuration, minimal preprocessing and postprocessing, and enhanced precision [91]. When compared to the traditional methods like BEM and FEM, the meshless approach substantially reduces the labor-intensive and time-consuming task of model meshing. However, this comes at the expense of increased computational processing time and overhead [92,93]. It is noteworthy that the meshless method can be intricate for heterogeneous geometric torso models with varied electrical conductivity structures [91].

The numerical methods outlined in this section are tailored to address various forward problems. Notably, more than one method might be suitable for a specific problem, dependent on its intricate details and specific needs. We explore them further:

(1) Both BEM and FEM focus on the differential weak form. They emphasize a global adherence to the governing equation but realize this adherence at the elemental level. This means each constituent element aligns with the central governing equation, though it may not strictly conform at every single point within an element. Their adaptability allows for greater flexibility in addressing various problem structures.

(2) FDM uniquely approaches the integral strong form, ensuring the governing equation’s fidelity at designated discrete points. However, this precision comes with a nuance: there may not be consistent adherence between these points. While this method offers pinpoint accuracy at these locations, it demands careful selection to ensure comprehensive representation of the entire problem space.

(3) It is worth noting that both FDM and meshless methods bypass the typical mesh partitioning, a staple in many numerical techniques. While initially appearing as a simplification, this choice can be two-sided. Eliminating mesh partitioning can enhance certain processes’ efficiency, but it often adds significant computational demands. Thus, while they might offer rapid results under some conditions, they can be resource-intensive, which may be a constraint for extensive or real-time simulations.

### 2.6. Challenges in Solving the Forward Problem

**Multiscale:** The cardiac forward modeling is a multiscale electrophysiological problem. At present, one can measure or simulate the transmembrane potential of cardiomyocyte, or even a single current through a single channel of the cell membrane [62,94]. However, when the goal is to capture the behavior of the entire heart, it becomes intractable to handle because there are thousands of ion channels in a myocyte, and billions of myocytes in the heart. In the future, forward modeling will begin at the subcellular level, starting with ion channels, progressing through cells and tissue blocks, and ultimately encompassing the entire heart and torso, with the aim of generating predictive clinical electrocardiograms. With rapid advances in biomedicine, imaging and computing, this may usher in a revolution. The challenge is therefore evident: to integrate the wealth of information in the relevant fields, enabling the determination of structure and function across all tiers of biological organization.

**Computability:** It is also necessary to consider the computability of the model, while dealing with the complex problem of multiscale modeling. Usually, numerical algorithms and computer resources always limit the level of detail that a model can contain, so the model needs to be properly simplified. Earlier models used a dipole to represent the heart source, and the model only needed to solve a simple set of linear equations [49]. As computers became more powerful, and source models included more content, we were able to simulate electrocardiograms in small animals [95,96] as well as humans [55,97,98]. Prior studies have demonstrated end-to-end forward simulations, from cellular electrophysiology to body-surface potentials, by coupling the bidomain model with biophysically realistic membrane dynamics [6,7,68]. To accommodate increasing model complexity while maintaining computational feasibility, researchers have leveraged more powerful computers and expanded the content of source models, having dedicated substantial effort to these advances [99,100,101,102]. Even so, current models have to use lower resolution and potentially incomplete approaches to be compatible with issues such as computability of the forward ECGI problem [103].

**Parameter Specificity:** For a specific research objective, choosing the most appropriate models and parameters is always an open challenge. The reaction–diffusion equation, for example, can be combined with a wide range of cell models that have slightly different action potentials, which in turn produce quantitatively or qualitatively different simulated electrocardiograms. Similarly, the results obtained by using different numerical methods will be different. Across all scales, there is variability and uncertainty in the problem, which is bound to have an impact on the accuracy of the solution.

For clinical patients, it has specific geometric data of the body and heart, cardiac pathology information (scar tissue, etc.), and tissue conductivity [104]. The influence of clinical cardiac pathology on the forward problem has always been a hot topic. In order to construct a specific forward model, at the cellular level, appropriate and specific parameters must first be determined to control the state of ion channels and the action potential of cells. Further, it is necessary to determine the types of cells that the heart contains and their distribution, each with different conductivity and action potential characteristics. In addition, the numerous tissues contained in the torso also contribute to the formation of body-surface potentials. It is not yet clear what level of accuracy or fidelity the conductivity parameters need to achieve in order to perform useful forward simulations. In short, fully adapting clinical patient-specific parameters is a challenge.

**Verifiability**: The implementation of ECGI is strongly dependent on the choice of cardiac source models and numerical methods. Validation methods need to consider these two components, which often present a formidable challenge. Through theoretical analysis, it has been established that regularization, based on physical and physiological prior information, substantially enhances the solvability and robustness of inverse solutions in electrocardiographic recordings. However, current techniques make it difficult to obtain highly detailed and locally invasive ground-truth data in living animals or humans. In clinical applications, the electrical activity of the heart does not occur in isolation; many factors, such as cardiac mechanics, coronary blood flow, the autonomic nervous system, etc., can significantly affect it. Coupled with the specificity of the patient, the validation of the forward model becomes increasingly difficult.

## 3. Current Techniques and Solutions

When faced with a heart containing billions of cells, it is not feasible to simulate detailed membrane behavior models of individual cells. To obtain a tractable forward solution, the heart’s multiscale physiology must be simplified at each relevant scale. Accordingly, the representation of the heart source, simplification strategy, and numerical method should be tailored to the simulation objective. In this section, we give the detailed modeling process of three common models, and make a comparative analysis of the models.

### 3.1. Cardiac Surface Potential (CSP) Models

The classic model of the forward problem hinges on a CSP model, wherein the cardiac source is represented by the extracellular electrical potential on the epicardial surface. Typically, this surface encompasses either the ventricles or atrial cavities, with a preference for using the epicardial surface to encompass them. For computational purposes, this surface is artificially closed off at the valves and the base, which forms a closed surface [26]. In the 1970s, Barr and his colleagues addressed this intricate issue, devising numerical solutions through boundary element methods [80]. Below is a concise overview of the CSP model.

**Figure 3:**
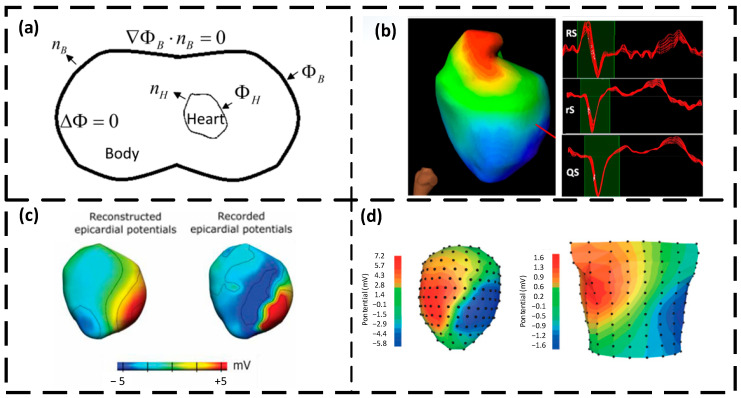
Applications of CSP model: (**a**) Schematic diagram of a surface potential model. (**b**) The ventricular surface potential and corresponding EGM reconstructed by Cardio Insight [105]. (**c**) The comparison of recorded epicardial potentials and reconstructed epicardial potentials from the CSP model [26]. (**d**) The input (epicardial potentials) and output (BSPs) of the CSP model [29].

Given that the cardiac source is uniformly distributed across the heart’s surface, and the space between the heart and the body surface acts as a homogeneous and isotropic volume conductor with a conductivity value (σ), the potential distribution within this conductor fluctuates over time and space. However, effects from electromagnetic propagation are not considered. Focusing on the potential distribution resulting from a time-varying current source within the volume conductor, this forward problem can be approached as a quasi-static problem. Within the context of the surface potential model, the forward problem calculates the body-surface potential $\Phi_{B}$ based on cardiac surface potential $\Phi_{H}$. As shown in Figure 3a, the human torso (represented by a volume conductor body) is encased within a closed surface $S_{B}$. This is enveloped by non-conductive air. Heart denotes the heart region, with $S_{H}$ representing the epicardial surface, which contains all bioelectric sources; ${\vec{n}}_{B}$ and ${\vec{n}}_{H}$ symbolize the unit normal vectors for the smooth surfaces $S_{B}$ and $S_{H}$ with an outward direction.

The quasi-static approximation of Maxwell’s equation adequately describes such a system, which is well approximated as linear, piecewise uniform, and isotropic [80,106]. For any field point p in volume conductor, the electric potential ϕ(p) satisfies the Laplace equation:

(6a) $$\sigma \nabla^{2}\phi (p)=0,p\in B$$

(6b) $$\sigma \nabla \phi (p)\cdot \vec{n_{B}}=0,\;p\in S_{B}$$

#### 3.1.1. Transfer Matrices of CSP Models

Using the boundary element method, Barr et al. give a detailed derivation process [80,107]. Equation (6) is transformed into an equivalent boundary integral equation for the potential ϕ(p) at any field point p by using Green’s second equation.

(7) $$c(p)\phi (p)=\int_{S_{B}}\phi_{B}\nabla \frac{1}{r}\cdot \vec{n_{B}}dS-\int_{S_{H}}\phi_{H}\nabla \frac{1}{r}\cdot \vec{n_{H}}dS-\int_{S_{H}}\frac{1}{r}\nabla \phi_{H}\cdot \vec{n_{H}}dS$$

where c(p)=2π for p inside B, and c(p)=4π for $p\in S_{H}\cup S_{B}$ [106]; dS=dS(q) is the differential of the integration surface; $\nabla r^{-1}\cdot \vec{n}dS=d\Omega$ is the solid angle, with r=q−p being the directed distance from the field point p to the source point q and r=|r|.

Observation points are placed on $S_{H}$ and $S_{B}$ respectively, to rewrite Equation (7), and the two equations for the locations of the observer give

(8a) $$\frac{1}{2\pi }\int_{S_{B}^{-}}\phi_{B}d\Omega_{BB}^{i}-\frac{1}{2\pi }\int_{S_{H}}\phi_{H}d\Omega_{BH}^{i}-\frac{1}{2\pi }\int_{S_{H}}\frac{1}{r^{i}}\nabla \phi_{H}\cdot \vec{n_{H}}dS-\phi_{B}^{i}=0$$

(8b) $$\frac{1}{2\pi }\int_{S_{B}}\phi_{B}d\Omega_{HB}^{i}-\frac{1}{2\pi }\int_{S_{H}^{-}}\phi_{H}d\Omega_{HH}^{i}-\frac{1}{2\pi }\int_{S_{H}^{-}}\frac{1}{r^{i}}\nabla \phi_{H}\cdot \vec{n_{H}}dS-\phi_{H}^{i}=0$$

where $S_{H}^{-}$ and $S_{B}^{-}$ denote integration over the surfaces $S_{H}$ and $S_{B}$, with the singularity removed; $r^{i}=q-p^{i}$ and $d\Omega_{PQ}^{i}$ refer to the solid angle subtended by the elemental area of integration over the surface Q at an observation point i on the surface P.

By converting the integral form to the discretized form, the potential on the body surface can be related to the potential on the heart surface; thus,

(9a) $$\sum_{j=1}^{N_{B}}p_{BB}^{ij}\phi_{B}^{j}+\sum_{j=1}^{N_{H}}p_{BH}^{ij}\phi_{H}^{j}+\sum_{j=1}^{N_{H}}g_{BH}^{ij}\Gamma_{H}^{j}=0$$

(9b) $$\sum_{j=1}^{N_{B}}p_{HB}^{ij}\phi_{B}^{j}+\sum_{j=1}^{N_{H}}p_{HH}^{ij}\phi_{H}^{j}+\sum_{j=1}^{N_{H}}g_{HH}^{ij}\Gamma_{H}^{j}=0$$

$N_{B}$ and $N_{H}$ represent the number of discrete grids on the body surface and the heart surface respectively. $\phi_{B}^{j}$ and $\phi_{H}^{j}$ are vectors containing the potential at the j locations on the surfaces of $S_{B}$ and $S_{H}$, respectively, and $\Gamma_{H}^{j}=\nabla \phi_{H}^{j}\cdot \vec{n_{H}}$ contains the normal component of the gradient on $S_{H}$. The coefficients p and g depend only on the geometry of $S_{B}$ and $S_{H}$ whose first superscript and first subscript identify the location and surface on which the observer is stationed, and the second superscript and subscript identify an element in the corresponding integral surface.

Equation (9) can be expressed in matrix notation as

(10a) $$P_{HB}\Phi_{B}+P_{HH}\Phi_{H}+G_{HH}\Gamma_{H}=0$$

(10b) $$P_{BB}\Phi_{B}+P_{BH}\Phi_{H}+G_{BH}\Gamma_{H}=0$$

Each *P* and *G* is a coefficient matrix completely dependent on geometry, with rows corresponding to different surface locations of the observer and columns corresponding to different locations of the integral surface.

The approach to solving Equation (10a) for $\Gamma_{H}=-G_{HH}^{-1}(P_{HB}\Phi_{B}+P_{HH}\Phi_{H})$ and then substituting the result into Equation (10b) to obtain the system [80] is as follows:

(11) $$\Phi_{B}=A\Phi_{H}={\left(P_{BB}-G_{BH}G_{HH}^{-1}P_{HB}\right)}^{-1}(G_{BH}G_{HH}^{-1}P_{HH}-P_{BH})\Phi_{H}$$

where A is the transfer coefficient matrix that directly relates the heart-surface potentials to body-surface potentials. Once the matrix has been calculated, the forward problem can be solved by converting heart-surface potentials into body-surface potentials using simple matrix multiplication. The detailed process of determining the elements of the P and G matrices from the thorax-heart geometry is shown in [80,106]. Various numerical methods, including FEM [108], BEM and MFS [109], were employed to solve the transfer matrix A, albeit in slightly distinct forms.

#### 3.1.2. Research Progress on CSP Models

Through numerical calculation of the inverse problem, the CSP model can directly provide potential maps and electrograms. The potential maps show the potential of the entire heart surface at a given time, and the electrograms show the change in potential of a single point on the heart surface over time. By reconstructing the heart-surface potential using this method, additional information can also be obtained, such as activation and recovery time, low amplitude potential, step potential, etc. [26,110]. In addition, an important limitation of the CSP model is the loss of electrophysiological information related to the electrical activity of the heart on the endocardium, particularly in the ventricular septum and atrial septum. Therefore, most studies only provide epicardial reconstruction, and some approaches aim to reconstruct endocardial potential simultaneously, although the feasibility of this approach is still under discussion [108]. The inverse problem of simultaneous epicardial and endocardium reconstruction becomes worse because the non-convex geometry (endocardial and epicardial surface) to be treated is more complex [111]. In contrast, the reconstruction of only epicardial potentials requires the relatively simple “convex hull” (epicardium or pericardium) [110]. Figure 3b–d show several examples of using the CSP model.

In recent decades, a large number of researchers have studied the optimization of ECGI based on the CSP model [112,113,114]. With the significant progress in numerical algorithms of inverse problems, the clinical application of the epicardial potential method in ECGI is increasing [115]. This approach has been successfully used to optimize cardiac resynchronization therapy, to detect macroscopic reentry circuits and electrical rotors in patients with atrial flutter, atrial fibrillation and reentrant ventricular tachycardia, and to guide catheter ablation of the origin of focal ventricular tachycardia [116,117]. In 2017, Cuculich et al. first reported the use of ECGI-guided noninvasive ablation for the treatment of human ventricular tachycardia [118]. In 2019, Kalinin et al. utilized a single layer source replacement potential model on the surface of the myocardium to make some physiological sense of the inverse problem and improve endocardial reconstruction [2].

### 3.2. Equivalent Double-Layer (EDL) Models

The EDL model uses the cardiac surface current dipole layer to represent the exocardiac potential generated by the volumetric electric source distribution in the myocardium. The current dipole layer includes an inner cavity and has a strength proportional to the transmembrane potential on the surface [119]. The EDL model can directly obtain the local activation or recovery time of the heart, distinguish the endocardial or epicardial activation, and then describe the polarization and repolarization of the heart [120,121]. The activation time is when the depolarized phase of the action potential arrives, and the recovery time is when the repolarization phase occurs. The EDL model is derived from the classical bioelectric double-layer model described by Wilson et al. [38], that is, the equivalent source of current generated at the boundary between active and stationary cells during depolarization. This current dipole layer model is used to describe the activity of depolarized wave front propagating through the heart muscle [26]. Later, Salu demonstrated the equivalence between the external electrical potential generated by the ventricular myocardium and the uniform dipole layer on the surface of the boundary myocardium [122]. Barnard et al. gave the basic derivation of the EDL model [123]. The primary limitation of the equivalent double-layer (EDL) approach lies in the mathematical singularity that arises in the computed potentials in close proximity to the dipole source, which may lead to unrealistic infinite values. Its main advantage, however, is the conceptual clarity and interpretability it offers, as it provides an intuitive link between the underlying bioelectric sources and the resulting extracellular potential field.

The basic assumption of the EDL model is that the exocardiac potential generated by the source distribution in the ventricular myocardium is equal to the potential generated by the dipole layer on the ventricular surface. Other assumptions are similar to those of the CSP model mentioned earlier. The volume conductor is isotropic with the conductivity σ, and the basic dipole current sources are evenly distributed on the surfaces of the heart. Based on activation/recovery time, the basic equation of the EDL model directly provides the body-surface potential ϕ(p,t) generated at a point p at time t.

(12) $$\phi (p,t)=\int_{S_{H}}K(p,q)H(t-\tau (q))dS_{q}$$

where K(p,q) is the transfer matrix of the potential at point p on the body surface, which is generated by an elementary double-layer source at point q on the ventricular surface $S_{H}$. H(t−τ(q)) is the Heaviside step function approximating the actual ventricular action potential; τ(q) represents the activation/recovery time of a point of q.

#### 3.2.1. Transfer Matrices of EDL Models

Barnard et al. give detailed derivation of the transition matrix [9,123]. Starting from the potential $U_{\infty }(p)$ at the field point p, generated by the elementary dipole source density $\vec{J_{i}}$ in an infinitely homogeneous conducting medium with a conductivity of *σ*,

(13) $$U_{\infty }(p)=\frac{1}{4\pi \sigma }\int_{V}\vec{J_{i}}\cdot \nabla \frac{1}{r}dV$$

Applying Green’s formula and Gauss’s theorem, the actual potential K(p,q) can be calculated by the following integral formula [124,125]:

(14) $$K(p,q)=2U_{\infty }(p,q)+\frac{1}{2\pi }\int_{S_{B}}K(p,q)d\Omega_{BB}$$

where $d\Omega_{BB}$ denotes the solid angle subtended by a differential of the integration at points p on the body surface. For all potential points $p_{i}$ and source points $q_{j}$ there are

(15) $$A=A_{ij}=K(p_{i},q_{j})$$

A is the desired transition matrix, representing the potential at $p_{i}$ due to an elementary source at $q_{j}$, $i\leq N_{B}$ and $j\leq N_{H}$. Thus, $A_{ij}$ is given by

(16) $$A_{ij}-\sum_{k=1}^{N_{B}}p_{BB}^{ik}A_{kj}\approx 2U_{\infty }(p_{i},q_{j})$$

where $p_{BB}^{ik}$ denotes the solid angle subtended by a differential of the integration surface $S_{B}$ at the $i_{th}$ node $p_{i}$ on the surface $S_{B}$. Further simplification leads to

(17) $$A=2{\left(I-P_{BB}\right)}^{-1}U$$

Note that singular value problems need to be considered [9,126].

#### 3.2.2. Research Progress on EDL Models

The EDL model can represent the total electrical activity of the atrium or ventricle at any given time point [127]. The EDL model is designed to obtain local polarization or repolarization time directly, without reconstructing the extracellular electrical potential or the transmembrane voltage as intermediate steps. Geselowitz’s study showed that the strength of the myocardial surface dipole layer, which represents the actual current source distribution in the heart, is proportional to the transmembrane voltage [128,129]. Generally, the source parameters of EDL models include at least two parameters, activation time and recovery time, and there are also complex versions with up to seven parameters [55]. The relationship between the source parameters and the source strength of these models is nonlinear. This nonlinearity means that the parameters cannot be directly transferred across different scenarios, so they need to be re-estimated for different applications [26].

**Figure 4:**
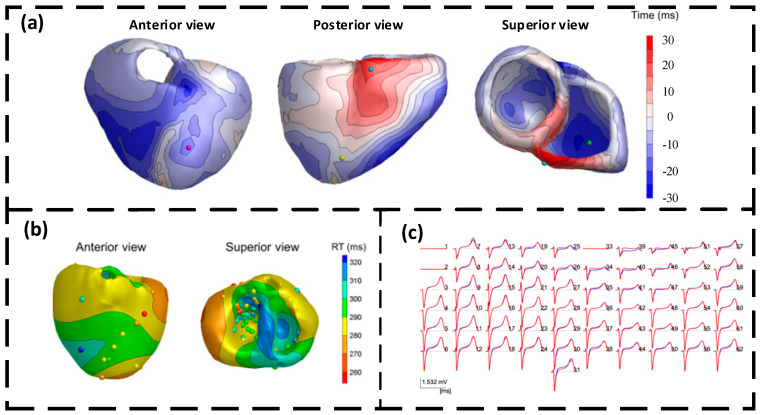
Some examples of applying EDL model. (**a**) Reconstruction of the ventricular endocardium and epicardium polarization times [5]. (**b**) The input of EDL model: anterior and superior views of the heart (with spheres indicating electrode and color for RT). (**c**) Superimposed measured (blue) and reconstructed (red) body-surface ECGs [4].

The cardiac polarization times derived from the EDL model have been demonstrated to be accurate, while the validation of repolarization times remains relatively insufficient. Figure 4 shows several examples of using the EDL model. Van der Waal, JG et al. confirmed the accuracy of the reconstructed cardiac repolarization time in the EDL model [5]. The reconstructed repolarization times exhibited a strong correlation with the gold standard (COR > 0.85). Moreover, this correlation remained nearly unchanged even after introducing tissue-conductivity errors or surface electrocardiographic noise into the model. Recently, Van der Waal, JG et al. used the EDL model to noninvasively estimate endocardial and epicardial polarization and repolarization time [4]. Their aim was to assess the temporal and spatial accuracy of the EDL method in reconstructing the repolarization times with increased repolarization heterogeneity, and they validated the EDL method on pig hearts. The results showed that EDL-based noninvasive ECG repolarization imaging of atrial and ventricular rhythms allows for the quantitative reconstruction of the repolarization change area.

Based on the EDL model, the clinical and simulation results of Zhou et al. show that the Bayesian method is feasible and accurate in locating left ventricular endocardial activation [119]. To achieve risk stratification of sudden cardiac death in patients with cardiomyopathy, Roudijk, RW et al. proposed a new inverse ECGI technique based on the EDL model, which can estimate the ventricular activation sequences of the endocardium and epicardium, further improving the consistency of the results [130]. To reduce the error caused by cardiac source under-sampling, Tate, JD et al. tested the EDL model forward simulations with different cardiac source resolutions and spatial interpolation techniques, and provided guidelines [131]. Janssen, AM et al. found that when using EDL models with an anisotropic heart model, almost all considered activation modes have significant localization errors, with an average positioning error of 20.4 mm [132]. These findings suggest that, in EDL-based activation time imaging using simulated BSPMs generated from an anisotropic finite element heart–torso model, cardiac anisotropy may have a limited marginal effect on certain reconstruction endpoints. However, this conclusion should not be generalized to all EDL-based clinical applications without further validation. More broadly, ECGI has been explored for characterizing ventricular activation and electrical dyssynchrony in patients considered for cardiac resynchronization therapy [115]. In 2024, Job Stoks shows that the activation and recovery patterns vary profoundly between normal subjects, but are stable individually beat to beat [133]. The feasibility of EDL in the forward model was verified in this research.

Similar to the EDL model, there is a class of 3D cardiac electrical imaging (3DCEI) models that are also based on activation and recovery time, which can reconstruct the activation/recovery process of the entire myocardium, including the epicardium, endocardial, and intramural tissues [134,135]. The main applications of the 3DCEI model are imaging ventricular activation sequences and reverse-reconstructing the equivalent current density [136,137], which has been extensively evaluated and mapped in some organisms [138,139,140,141]. At present, the 3DCEI model may be more suitable for the electroencephalogram field.

### 3.3. Transmembrane Voltage (TMV) Models

TMV models use the potential difference of cardiomyocytes across the cell membrane as the cardiac source model. This model is constrained by the electrophysiological activity in the real myocardium, and is the approximation closest to the real cardiac electrical source supply at present. Compared with the CSP and EDL models, the TMV model can realize not only endocardial and epicardial surface reconstruction but also transmural reconstruction [100,142]. TMV models are based on the bidomain model, which is closely combined with the electrophysiological model of cardiomyocytes, and can take into account the anisotropy of the electrical conductivity of the myocardium region [143]. In clinical environments, because the TMV model cannot be directly available, the TMV distribution needs to be translated into extracellular electrical potential on endocardial and epicardial surfaces or activation/recovery times before being validated. The following gives the basic derivation of the TMV model.

In order to reduce the complexity of the model, the torso can be assumed as a volume conductor, the induction and displacement currents in the tissue are ignored, and the field calculation can be effectively described by a quasi-static approximation based on Maxwell’s equations. Within the myocardium region $\Omega_{H}$, based on the bidomain theory, the governing Equation (18a) can be expressed by a second-order elliptic differential equation. In the region $\Omega_{T/H}$ between the surface of the heart and the surface of the body, assuming no other active electrical source exists, the governing Equation (18b) can be expressed by Laplace’s equation.

(18a) $$\nabla (\kappa_{H}(p)\nabla \phi_{e}(p))=-\nabla (\kappa_{i}(p)\nabla u(p)),\;\forall p\in \Omega_{H}$$

(18b) $$\nabla (\kappa_{T}(p)\nabla \phi_{T}(p))=0,\forall p\in \Omega_{T/H}$$

where $\kappa_{H}$ is the overall effective conductivity tensor of the heart, $\kappa_{H}=\kappa_{i}+\kappa_{e}$. In the bidomain theory, $\kappa_{i}$ and $\kappa_{e}$ are the effective intracellular and extracellular conductivity tensors. Equation (18a) describes the relationship between the transmembrane voltage u(p) and the extracellular potential $\phi_{e}(p)$ at the space point p. In formula (18b), $\kappa_{T}$ and $\phi_{T}$ refer to the torso conductivity tensor and potentials.

#### 3.3.1. Myocardial Transmembrane Potential Activity Model

The complexity of cardiac electrophysiological models ranges from two-variable equations at the macro level to the Luo–Rudy model [144] at the cellular level, with more than 15 variables [100]. Considering model plausibility with computational feasibility, a two-variable system is favorable in the IECG study, which is represented by the FitzHugh–Nagumo model [69,70]. Equation (5) gives the basic form of the FitzHugh–Nagumo model, and Equation (19) provides the specific forms of $f_{1}(u,v)$ and $f_{2}(u,v)$.

(19) $$\begin{array}{l} f_{1}(u,v)=c_{1}u(u-\alpha )(1-u)-c_{2}v,\\ f_{2}(u,v)=b(u-dv) \end{array}$$

where α, b, d $c_{1}$ and $c_{2}$ are “membrane” parameters, defining the shape of the action potential pulse, α is the dimensionless threshold voltage, b is the resume rate constant, d is the attenuation constant, $c_{1}$ is the excitation rate constant and $c_{2}$ is the excitation attenuation constant.

Variations of $f_{1}(u,v)$ and $f_{2}(u,v)$ produce different action potential shapes. To provide a more cardiac-like waveform, $f_{1}(u,v)$ was modified slightly by Rogers and McCulloch [145].

(20) $$f_{1}(u,v)=c_{1}u(u-\alpha )(1-u)-c_{2}uv$$

In addition, Rogers and McCulloch give recommended values for model parameters [145] α = 0.13, b = 0.013, d = 1.0, $c_{1}$ = 0.26 and $c_{2}$ = 0.1. In anisotropic simulations, the diffusion tensor D has a value of 1 along the muscle fiber and 4 along the transverse direction. In isotropic simulations, D = 1.

To accurately capture the dynamics of pulse propagation in canine myocardium, Aliev and Panfilov modified the excitable medium of the FitzHugh–Nagumo model using the following equations [144]:

(21) $$\begin{array}{l} f_{1}(u,v)=cu(u-\alpha )(1-u)-uv,\\ f_{2}(u,v)=\varepsilon (u,v)(-v-cu(u-\alpha -1)) \end{array}$$

where $\varepsilon (u,v)=\varepsilon_{0}+\mu_{1}v/(u+\mu_{2})$, α = 0.15, c = 8, $\varepsilon_{0}$ = 0.002, $\mu_{1}$ = 0.2, and $\mu_{2}$ = 0.3. ε(u,v) controls the coupling between the transmembrane action potential and the recovery current, α controls cell excitability and c controls repolarization.

For more detailed transmembrane potential models, parameterization is usually based on experimentally measured or previously published ionic models. Hodgkin–Huxley-type and Luo–Rudy-type models, for example, require parameters such as membrane capacitance, maximum ionic conductances, reversal potentials, ionic concentrations, and gating-variable kinetics. These parameters determine the shape, duration, and restitution properties of the action potential. At the tissue scale, propagation models such as the monodomain and bidomain equations introduce additional parameters, including diffusion coefficients or intracellular and extracellular conductivity tensors, myocardial fiber orientation, membrane surface-to-volume ratio, and stimulation settings. These parameters directly affect conduction velocity, wave front curvature, activation time, repolarization sequence, and ultimately the body-surface potentials computed by the forward model. In practice, model parameters are commonly obtained from experimental electrophysiological measurements, established reference models, or calibration against measured ECGs, activation maps, or body-surface potential maps. Because different parameter choices can lead to different activation patterns, repolarization sequences, and ECG morphologies, parameter selection and sensitivity analysis are important steps in ECGI forward simulations.

#### 3.3.2. Transfer Matrices of TMVs

**BEM-FEM:** Fischer, G. et al. solved the problem using the BEM-FEM coupling technique [143]. The FEM is used for the heart region $\Omega_{H}$, and according to Equation (18a), it can be obtained as follows:

(22a) $$\int_{\Omega_{H}}\nabla n_{i}\cdot \kappa_{H}\nabla \phi d\Omega -\oint_{S_{H}}n_{i}J_{n}(p)\cdot dS=-\int_{\Omega_{H}}\nabla n_{i}\cdot \kappa_{i}\nabla ud\Omega$$

(22b) $$J_{n}(p)=-[\kappa_{H}\nabla \phi (p)+\kappa_{i}\nabla u(p)]\cdot \vec{n_{H}},\;\forall p\in S_{H}$$

where $n_{i}$ is the finite element basis function. ϕ and u indicate the appropriate node potential. $S_{H}$ is the boundary of the heart region $\Omega_{H}$, and $n_{H}$ is the normal quantity of $S_{H}$. Formula (22a) is a common form of the finite element method where the surface integral is related to the normal of current density $J_{n}$ across the boundary $S_{H}$.

The BEM is used for the torso region $\Omega_{T/H}$. According to (18b), it can be seen that

(23a) $$c(p)\phi (p)+\oint_{S_{B}}\phi \nabla \frac{1}{r}\cdot \vec{n_{B}}dS-\int_{S_{B}}\frac{1}{r}\nabla \phi \cdot \vec{n_{B}}dS=0,\forall p\in S_{B}$$

(23b) $$J_{n}(p)=\kappa_{T}\nabla \phi (p)\cdot \vec{n_{B}},\forall p\in S_{B}$$

Equation (23a) is a standard boundary integral equation that applies to all field computation problems based on Laplace’s equations, where p denotes the field point, q is the source point, and r=|p−q| and c(p) refer to the interior solid angle at point p. Equation (23b) describes the normal component of the current density across $S_{B}$.

On the coupling interface, the number of nodes and the node positions must be equal in the finite element mesh and boundary element mesh. The coupling boundary must satisfy the condition that the tangential component of the electric field is continuous, which is represented by selecting a continuous potential, and that the normal component of the current density must be continuous.

(24) $$J_{n}(p)=-[\kappa_{H}\nabla \phi (p)+\kappa_{i}\nabla u(p)]\cdot \vec{n_{H}}=\kappa_{T}\nabla \phi (p)\cdot \vec{n_{B}}$$

By discretization, (22a) and (23a) are converted into matrix form

(25a) $$D_{H}\phi^{H}-T_{H}J=-D_{i}U$$

(25b) $$\kappa_{T}H\phi^{B}-GJ=0$$

where $D_{H}$ and $D_{i}$ are FEM stiffness matrices that consider the anisotropy properties of intracellular and extracellular space. $T_{H}$ and U are called the coupling matrix and transmembrane potentials. $\phi^{H}$ and $\phi^{B}$ represent the electric potential at all finite element and boundary element nodes, respectively. G and H are called single- and double-layer matrices. J contains the normal component of current density at all coupling nodes, which is obtained by eliminating it:

(26) $$(D_{H}-\kappa_{T}T_{H}G^{-1}H)\phi =-D_{i}U$$

The method of elimination by extracting j is often referred to as BEM-FEM coupling, and there is a more detailed procedure [146,147].

**BEM–Meshless:** Lin Wei et al. used mesh-free points to represent myocardium regions [100]. In order to reduce the complexity of the model, only the anisotropy of $\kappa_{i}$ is retained [148]. In the isotropic and homogeneous volume conductor of the torso $\Omega_{T}$, Formula (18) can be simplified as

(27) $$\sigma \nabla^{2}\phi (p)=\nabla (\kappa_{i}(p)\nabla u(p)),\forall p\in \Omega_{T}$$

By using the direct method solution of BEM [149], the following can be obtained:

(28) $$c(p)\phi (p)+\int_{S_{T}}\phi (q)\xi^{*}(p,q)dS-\int_{S_{T}}\frac{\partial \phi (q)}{\partial \vec{n}}\phi^{*}(p,q)dS=\int_{\Omega_{T}}\frac{\nabla \kappa_{i}\nabla u(q)}{\sigma }\phi^{*}(p,q)d\Omega$$

where $S_{T}$ is the boundary of $\Omega_{T}$, and $\vec{n}$ is the outward normal vector of the boundary surface. $\phi^{*}(p,q)$ and $\xi^{*}(p,q)$ are the so-called fundamental solution and its normal derivative [149].

Using the meshless strategy, the right side of Equation (28) (the volume integral part) can be simplified as

(29) $$\int_{\Omega_{T}}\frac{\nabla \kappa_{i}\nabla u(q)}{\sigma }\phi^{*}(p,q)d\Omega =\int_{S_{T}}\frac{\phi^{*}(p,q)}{\sigma }\kappa_{i}\frac{\partial u(q)}{\partial \vec{n}}dS-\int_{\Omega_{T}}\frac{\nabla \phi^{*}(p,q)}{\sigma }\kappa_{i}\nabla u(q)d\Omega$$

Assuming that neither the active current nor the passive current leaves the surface $S_{T}$, it can be further simplified as

(30) $$c(p)\phi (p)+\int_{S_{T}}\phi (q)\xi^{*}(p,q)dS=-\int_{\Omega_{T}}\frac{\nabla \phi^{*}(p,q)}{\sigma }\kappa_{i}\nabla u(q)d\Omega$$

The boundary integral and the volume integral use the BEM and meshless strategy respectively, and Equation (30) can be converted into the following matrix expression:

(31) LΦ=BU

where matrices L and B contain the geometrical and conductivity information in personalized heart–torso structures. By matrix operations, Equation (31) can be converted to the form Φ=HU. The transition matrix H is very sensitive to perturbations of solutions, and the inverse problem of Equation (31) is seriously ill-posed. Lin Wei et al. used the cardiac electrophysiological model as prior knowledge to constrain the model [100].

#### 3.3.3. Research Progress on TMV Models

**Figure 5:**
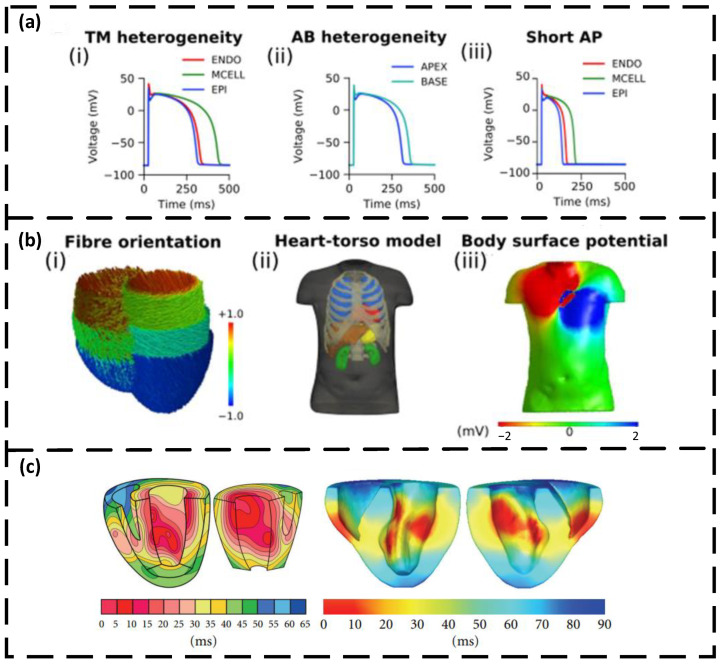
Some examples of applying TMV model. (**a**) Transmembrane action potentials (the source of cardiac electrical activity): (**i**) transmural (TM) heterogeneity in cells from the endocardium (ENDO), mid-myocardium (MCELL), and epicardium (EPI), (**ii**) apico-basal (AB) heterogeneity, and (**iii**) short single-cell action potentials. (**b**)The inputs and output of TMV model: (**i**) fiber orientation; (**ii**) heart–torso model; (**iii**) BSP, the output of the TMV model [153]. (**c**) Reconstruction of ventricular transmural action potentials [151].

Reconstruction of the TMV model can identify depolarizing and repolarizing areas of the heart, and can outline areas with reduced electrical signal amplitude or complete absence of electrical signals, such as ischemia, fibrosis, or marginal areas [26]. Like the EDL model, the TMV model can use prior electrophysiological knowledge of cardiomyocyte action potential propagation to constrain reconstruction [6]. It is worth noting that the TMV model provides the possibility for reconstruction through the ventricular wall [100,150], and also allows the endocardial and epicardial surfaces to be reconstructed simultaneously [142]. Figure 5 shows several examples of using the TMV model.

For the first time, Lin Wei et al. attempted to integrate physiological knowledge into a 3D myocardium model to reconstruct volumetric TMV dynamics in the 3D myocardium, and evaluated the robustness of the framework to real-world model and data errors [100]. The model employs a volumetric TMV activity model as a constraint for TMV imaging and uses Bayesian methods to account for uncertainties in the model and data. The model takes into account the anisotropy of the ventricular muscle. The localization error of endocardial lesions was less than 5.8 mm, and the mean localization error of intracardiac ectopic lesions was 4.5 mm. For the atria, Schuler et al. used the TMV model to propose a new method to improve the reconstruction of atrial ectopic activity, especially in scenarios with a low signal-to-noise ratio [7]. The method constructs a spatiotemporal basis of the body-surface potential and reconstructs a linear combination of the corresponding TMV basis vectors. This method is more robust and accurate in locating ectopic lesions. When SNR is 0 dB, TMV correlation is increased by 32%, the average local activation time correlation is increased by 19%, and the average localization error can be halved from 15.8 mm to 7.9 mm.

Zhang et al. used the element-free Galerkin method to simulate the propagation of myocardial electrical activation. They converted the TMV model based on the monodomain equation into the weak form of the Galerkin method and presented a solution technique [151]. This method is used to simulate the propagation of myocardial activation using a mesh-free particle representation. Potyagaylo et al. successfully reconstructed the activation pattern and origin of simulated extrasystole by applying a binary optimization method to the TMV model [152]. Zaman et al. used the posterior Bayesian active learning method to estimate the electrophysiological parameters of the heart in the TMV mode, and the experimental results showed that its performance was improved [6]. Dhamala et al. studied the spatial non-uniform resolution of myocardial tissue in the TMV model [72].

### 3.4. Comparative Analysis of Techniques

CSP model advantages: The potential-based model stands out due to its inherent benefits. It is simpler to obtain real clinical trial data for this model, making it highly clinically relevant among all cardiac models. Additionally, it often forms the foundation for some commercial systems [26,154,155]. While these models offer insights into the epicardial and endocardial surfaces, they cannot directly depict transmural propagation. This limitation arises as the heart source lacks detailed data on the cardiac action potential. Consequently, many ECGI implementations using the potential-based approach offer only epicardial reconstructions, excluding the endocardial or ventricular septal potentials. Furthermore, this model only focuses on the heart–torso geometry, excluding internal heart electrophysiological details. Table 1 summarizes the recent progress of three models.

EDL Model Advantages: The EDL model presents a clear edge over the potential model by utilizing prior physiological knowledge. When tackling the inverse problem, this model uses preliminary estimations of activation/recovery times and iteratively refines them, thereby enhancing accuracy and minimizing convergence time. Another merit of the EDL model is its ability to reconstruct the endocardium, though not directly into the myocardium. Current research is shifting towards algorithmic model estimation, especially concerning inhomogeneous cardiac activation, with such cases occurring due to scar tissues.

TMV Model Distinctions: The TMV model is the most robust, enabling reconstructions not just of the ventricular and atrial endocardial and epicardial membranes, but also the inner myocardium. The major strength of the TMV model lies in the use of prior knowledge of action potential propagation for model constraints. Given the clinical limitations in directly measuring TMV, validation entails computing extracellular electrical potentials on endocardial/epicardial surfaces and deriving activation/recovery times from the TMV distribution, followed by indirect validation against experimental measurements. Striking a balance between the TMV model’s adaptability and computational feasibility remains a challenge. Presently, studies on the TMV model mainly focus on the two-variable diffusion-response system [7,72,159], which represents a realm with significant potential for enhancing electrophysiological accuracy and information.

### 3.5. Atrial vs. Ventricular Forward Modeling

Although CSP, EDL, and TMV models can be formulated within a common ECGI forward-modeling framework, their implementation differs between atrial and ventricular applications. These differences arise from chamber-specific anatomy, electrophysiological properties, modeling objectives, and validation strategies [32].

In ventricular ECGI, CSP models are commonly defined on epicardial or combined epicardial–endocardial surfaces and are often used to describe ventricular activation, repolarization, pacing responses, and ventricular tachycardia. EDL models provide a compact surface–source representation for ventricular activation and recovery, while TMV-based models are particularly useful when the forward problem is coupled with volumetric electrophysiological simulations, tissue anisotropy, transmural propagation, or patient-specific digital-twin models [18]. In atrial ECGI, CSP and EDL formulations are frequently used for atrial activation mapping, ectopic-focus localization, P-wave modeling, and atrial fibrillation analysis [116,127]. TMV atrial models may also be used in mechanistic simulations, but their routine application in clinical ECGI is limited by the thin atrial wall, complex atrial anatomy, and sensitivity to segmentation and regularization assumptions. The typical applications and key considerations of CSP, EDL, and TMV source representations in atrial and ventricular ECGI forward modeling are summarized in Table 2.

Geometrical construction also differs between the two chambers. Ventricular models often require artificial closure near the basal or valvular planes to obtain closed epicardial or endocardial source surfaces [104]. In atrial models, however, source–surface construction is more anatomy-dependent because of the pulmonary veins, venae cavae, coronary sinus, and atrioventricular valve openings. These structures may be truncated, closed, or extended into vessel sleeves depending on the study objective, especially in left atrial modeling for atrial fibrillation or P-wave analysis.

Electrophysiological assumptions further distinguish atrial and ventricular forward modeling. Ventricular models must account for thick myocardial walls, transmural activation, Purkinje-mediated excitation, fiber anisotropy, and regional repolarization gradients, which are essential for QRS and T-wave generation as well as ventricular arrhythmia modeling. Atrial models generally involve thinner walls, shorter action potential duration, shorter refractory periods, and highly heterogeneous conduction around anatomical structures such as the pulmonary veins, atrial appendages, crista terminalis, and interatrial connections [44,160]. As a result, atrial ECGI studies often focus on activation timing, dominant-frequency regions, phase patterns, and driver localization, whereas ventricular ECGI more commonly emphasizes activation sequence, recovery time, transmural propagation, scar-related conduction delay, and ventricular tachycardia localization [3,116].

Validation strategies are also chamber-specific. Ventricular ECGI forward models are commonly assessed using epicardial sock recordings, endocardial or epicardial contact mapping, torso-tank experiments, pacing-site localization, or ventricular tachycardia exit-site comparison [25,157,161]. Atrial ECGI validation is more challenging because atrial signals are lower in amplitude and may overlap temporally with ventricular activity, often requiring QRST cancellation or dedicated atrial-signal extraction [162]. Therefore, atrial validation frequently relies on invasive atrial electroanatomical mapping, pacing or ectopic-focus localization, ablation targets, dominant-frequency mapping, or pulmonary-vein-related activation patterns.

**Table 2:** Typical applications of CSP, EDL, and TMV source representations in atrial and ventricular ECGI forward modeling.

Source Representation	Ventricular Applications	Atrial Applications	Key Considerations
CSP	Epicardial or epicardial–endocardial potential mapping [163]; ventricular activation and repolarization [164]; pacing-site or ventricular tachycardia localization.	Atrial activation mapping [165]; AF analysis [166]; ectopic-focus localization; atrial electrogram reconstruction [167].	Sensitive to source–surface geometry, electrode coverage, and regularization.
EDL	Compact representation of ventricular activation and recovery; QRS and T-wave modeling [168].	P-wave generation [127]; atrial activation modeling; simplified AF or atrial arrhythmia source descriptions.	Requires appropriate activation/recovery timing and chamber-specific surface closure.
TMV	Volumetric electrophysiological simulation; transmural activation; anisotropic propagation; ventricular digital-twin applications [169].	Mechanistic atrial simulations [170]; less common in routine clinical ECGI [171].	Physiologically interpretable but requires detailed geometry, conductivity, and electrophysiological parameters.

## 4. Public Datasets for ECGI Research

Public datasets are important for the reproducibility, validation, and comparison of ECGI studies. One of the most mature public resources is the Experimental Data and Geometric Analysis Repository (EDGAR), an open-access online repository developed by the Consortium for ECG Imaging to provide curated datasets for the application and validation of ECGI techniques [171]. EDGAR hosts heterogeneous ECGI-related datasets, including torso-tank experiments, animal and human mapping data, simulation datasets, anatomical geometries, and body-surface potential recordings.

These datasets are valuable for ECGI because they provide the key information required to construct and evaluate forward operators, such as heart–torso geometries, electrode locations, cardiac source measurements, and measured body-surface potentials. For example, EDGAR includes torso-tank and simulation datasets that can be used to test different source representations, numerical methods, source-sampling strategies, and geometric assumptions. A benchmark forward-problem study using an EDGAR dataset showed that complete cardiac source sampling substantially improves the agreement between computed and measured torso potentials, emphasizing the value of public datasets for validating ECGI forward models.

However, public datasets specifically designed for ECGI validation remain limited. Existing datasets vary in electrode configuration, anatomical detail, source sampling, conductivity assumptions, and available ground truth, which makes direct comparison across studies difficult. Future datasets should therefore provide standardized geometries, tissue labels, conductivity settings, electrode coordinates, source measurements, body-surface potentials, and benchmark evaluation metrics. Such resources would facilitate more reproducible and comparable studies of CSP, EDL, and TMV models.

## 5. Conclusions and Outlook

Unlike previous reviews that primarily focus on ECGI inverse reconstruction, clinical applications, or validation studies, this review emphasizes the forward problem from the perspective of mathematical source modeling. By systematically organizing ECGI forward modeling into three representative frameworks, namely cardiac surface potential (CSP), equivalent double-layer (EDL), and transmembrane voltage (TMV) models, we provide a unified view of how different source representations are formulated and how they determine the construction of the forward operator. This modeling-centered perspective helps clarify the theoretical basis of ECGI and highlights the central role of the forward problem in determining what cardiac electrophysiological information can be reconstructed from body-surface potentials.

In this review, we briefly summarized the modeling process of the ECGI forward problem, including cardiac source models, propagation models, volume conductors, numerical methods, and major challenges. We also presented the mathematical derivations and applications of CSP, EDL, and TMV models. In general, CSP models are relatively mature and computationally efficient, but they are mainly limited to surface-potential representations. EDL models provide a compact formulation for activation and recovery imaging, but their performance depends on assumptions about cardiac source distribution and model configuration. TMV models offer a more physiologically detailed description of cardiac electrical activity, but they also introduce higher computational complexity and greater dependence on model parameters.

Despite substantial progress, several unresolved bottlenecks remain in ECGI forward modeling. First, the construction of patient-specific heart–torso models is still affected by uncertainties in anatomical segmentation, electrode localization, tissue-conductivity assignment, and anisotropy modeling. These factors directly influence the forward operator and may affect the accuracy and robustness of inverse reconstruction. Second, the selection and parameterization of cardiac source models remain challenging. Although CSP, EDL, and TMV models provide different levels of physiological detail, there is still no unified criterion for selecting the most appropriate framework for a specific clinical or research task. Third, the balance between physiological realism and computational feasibility remains a central issue, especially for TMV-based and multiscale electrophysiological models. Finally, the lack of standardized public datasets, benchmark geometries, conductivity settings, and evaluation metrics limits reproducible comparison across different forward-modeling approaches.

Future ECGI forward models should therefore move toward patient-specific, disease-specific, computationally efficient, and experimentally validated frameworks. In particular, more attention should be paid to standardized model construction, reliable parameter assignment, systematic sensitivity analysis, and the development of larger publicly available clinical datasets and benchmark datasets. These efforts will help improve the reliability of ECGI forward modeling and support its broader application in the analysis of complex cardiac diseases such as myocardial infarction and atrial fibrillation. We hope that this review provides a useful mathematical and methodological reference for future academic and industrial research in ECGI.

## Figures and Tables

**Table 1:** A partial summary of 3 methods to solve the ECGI forward problem.

Core Methods	Refs.	Numerical Methods	Source Position	Clinical Data
**CSP**	Kalinin et al. [2]	FEM, BEM	Endocardium, epicardium of ventricle	Simulation only
	Pedron-Torrecilla et al. [3]	BEM	Epicardium of atrium	ECGs for four AF patients who underwent ablation; EGMs(Electrogram) recorded in two of them
	Jeanne van der Waal et al. [121]	FEM	Endocardium and epicardium of ventricle	EGMs of four ex vivo pig hearts
	Matthijs Cluitmans et al. [156]	BEM/FEM	Epicardium of whole heart	EGM of a PVC patient
	Yesim Serinagaoglu Dogrusoz et al. [58]	BEM	Endocardium and epicardium of ventricle	BSPs and EGMs of 10 PVC patients, and the EDGAR dataset
	B. Messnarz et al. [108]	BEM, FEM	Endocardium and epicardium of whole heart	EGM of a Wolff–Parkinson–White syndrome patient and an AF patient
	Laura R. Bear et al. [157]	BEM	Epicardium of ventricle	EGM and BSPs of ex vivo pig hearts in torso-tank
**EDL**	Jeanne van der Waal et al. [5]	BEM, FEM	Endocardium and epicardium of ventricle	Only simulation
	Shijie Zhou et al. [125]	BEM	Endocardium of LV	12-lead ECG of 38 arrhythmia patients who underwent left ventricular ablation
	Steffen Schuler et al. [7]	BEM	Epicardium of atrium	Only simulation
	Adriaan van Oosterom [120]	BEM	Endocardium and epicardium of ventricle	Only simulation
	Robert W. Roudijk et al. [130]	BEM	Endocardium and epicardium of ventricle	EAM of 13 patients who underwent ablation
	Jess D. Tate et al. [131]	BEM	Endocardium and epicardium of ventricle	Only simulation
	Arno M. Janssen et al. [132]	BEM	Endocardium and epicardium of ventricle	Only simulation
	Jeanne van der Waal et al. [4]	BEM	Endocardium, epicardium of ventricle	EGM of four Langendorff-perfused pig hearts
**TMV**	Linwei Wang et al. [100]	BEM, Mesh-free	Ventricle	123-lead BSPs of a post-MI patient and infarct location from cardiologists
	Mark Potse et al. [68]	BEM	Ventricle	Only simulation
	Qiang Zhang et al. [158]	BEM	Epicardium of whole heart	Only simulation
	G. Fischer et al. [143]	BEM, FEM	Ventricular	Only simulation
	Adriaan van Oosterom [148]	BEM	Whole heart	Only simulation
	Md Shakil Zaman et al. [6]	BEM	Ventricle	ECGs of three myocardial infarction patients who underwent ablation
	Rui Shi et al. [159]	BEM	Left atrium	EGM of human left atrium collected by AcQmap
	Heye Zhang et al. [151]	Mesh-free	Ventricle	Only simulation

## Data Availability

No new data were created or analyzed in this study.

## Abbreviations

The following abbreviations are used in this manuscript:

ECGI	Electrocardiographic imaging
CSP	Cardiac surface potential
EDL	Equivalent double-layer
TMV	Transmembrane voltage
CT	Computer tomography
MR	Magnetic resonance
BEM	Boundary element method
FEM	Finite element method
FVM	Finite Volume Method
FDM	Finite Difference Method
3DCEI	3D cardiac electrical imaging
TM	Transmural
AT	Activation time
RT	Repolarization time
ENDO	Endocardium
MCELL	Mid-myocardium
EPI	Epicardium
AB	Apico-basal
EGM	Electrogram
